# An Integrated Use of Topography with RSI in Gully Mapping, Shandong Peninsula, China

**DOI:** 10.1155/2014/827124

**Published:** 2014-08-05

**Authors:** Fuhong He, Tao Wang, Lijuan Gu, Tao Li, Weiguo Jiang, Hongbo Shao

**Affiliations:** ^1^College of Geography and Planning, Ludong University, Yantai 264025, China; ^2^Institute of Systems Science and Mathematics, NAAU, Yantai 264001, China; ^3^Hubei Environmental Science Academy, Wuhan 430072, China; ^4^State Key Laboratory of Earth Surface Processes and Resource Ecology, Beijing Normal University, Beijing 100875, China; ^5^Key Laboratory of Coastal Biology & Bioresources Utilization, Yantai Institute of Coastal Zone Research, Chinese Academy of Sciences (CAS), Yantai 264003, China; ^6^Jiangsu Academy of Agricultural Sciences, Nanjing 210014, China

## Abstract

Taking the Quickbird optical satellite imagery of the small watershed of Beiyanzigou valley of Qixia city, Shandong province, as the study data, we proposed a new method by using a fused image of topography with remote sensing imagery (RSI) to achieve a high precision interpretation of gully edge lines. The technique first transformed remote sensing imagery into HSV color space from RGB color space. Then the slope threshold values of gully edge line and gully thalweg were gained through field survey and the slope data were segmented using thresholding, respectively. Based on the fused image in combination with gully thalweg thresholding vectors, the gully thalweg thresholding vectors were amended. Lastly, the gully edge line might be interpreted based on the amended gully thalweg vectors, fused image, gully edge line thresholding vectors, and slope data. A testing region was selected in the study area to assess the accuracy. Then accuracy assessment of the gully information interpreted by both interpreting remote sensing imagery only and the fused image was performed using the deviation, kappa coefficient, and overall accuracy of error matrix. Compared with interpreting remote sensing imagery only, the overall accuracy and kappa coefficient are increased by 24.080% and 264.364%, respectively. The average deviations of gully head and gully edge line are reduced by 60.448% and 67.406%, respectively. The test results show the thematic and the positional accuracy of gully interpreted by new method are significantly higher. Finally, the error sources for interpretation accuracy by the two methods were analyzed.

## 1. Introduction

Gully erosion is a major contributor of sediment in streams and needs serious attention [1–5]. As a common type of soil erosion, due to the characteristics of significant volumes of sediment, fast speed, cutting the full slope into gullies with scattered small sloping areas and reducing arable land, gullies have caused very serious harm to agricultural production and have become a major source of river sediment [6, 7]. A series of studies recently indicate sediment yield caused by gully erosion accounted for 10% to 94% of the total watershed sediment yield [1].

A gully is a relatively deep, recently formed eroding channel existing on valley sides and on valley floors where no well-defined channel previously occurred [1, 8]. Gully edge line and gully thalweg are important topographical features for the study of gully erosion and topographical development. Gully edge line, the boundary between gully slope and inter-gully slope, is located in the most unstable parts of the gully. Gully thalweg is the boundary between gully floors and gully slope. Effectively interpreting and measuring gully edge line and thalweg will contribute to research on the development of gully erosion. Remote sensing technology has become an important tool for rapid extraction of gully [8–21]. However, currently there are still some problems in extracting gully edge line based on planimetric remote sensing images. (1) Although planimetric remote sensing images contain abundant spectral information, gullies do not differ generally from the surrounding environment in terms of spectral values. Therefore, interpreting gully edge line based on two-dimensional remote sensing images only has low accuracy. (2) Affected by factors such as topographic relief, sun elevation, the azimuth angle, the viewing angle, and hatching or engraving features present on the slopes at the time of imaging, there are significant differences in image radiance between the sunny and shadowed slopes which results in certain three-dimensional perception of the remote sensing images. However, the three-dimensional terrain information provided by planimetric remote sensing images is insufficient for precisely interpreting gully edge line. The integration of spectral information of the remote sensing images with three-dimensional terrain information to improve accuracy of interpreting gullies will certainly be conducive to the research of gully erosion.

The paper, taking the small watershed of Beiyanzikou valley of Qixia city, Shandong province, China, as the study area, proposed a new method to interpret gully edge line based on the fused image of terrain information and remote sensing images. Firstly, in the HSV color space, the simulated northwest illumination SRM data and *V* component data were summed with linear surface roughness *ϖ*′ and 1 − *ϖ*′ as weights, respectively, to build a new component *V*′. Then it was retransformed from HSV color space into RGB color space to construct fused image integrating remote sensing image spectral information with three-dimensional topographical information which accord to the human visual perception. Based on the topographic gradient threshold values field measured along gully thalweg and gully edge line, the slope data were segmented by thresholding to get gully thalweg and gully edge line threshold vectors, respectively. Then gully thalweg threshold vectors were amended based on visual interpretation in combination with gully thalweg threshold vectors, terrain, and fused image. Combined with amended gully thalweg vectors, slope gradient data, and fused image, gully edge line threshold vectors were amended. Lastly, a testing region was selected in the study area to make field measurements along gully head and gully edge line, and kappa coefficient and overall accuracy of error matrix and deviation were selected as evaluation indices to assess the thematic accuracy of the gully and the positional accuracy of gully head and gully edge line interpreted by both the method of interpreting remote sensing images only and fused image. The quantitative analysis showed that fused image improved 24.080% and 264.364% in overall accuracy and kappa coefficient compared with interpreting remote sensing images only. The average deviations of gully head are 2.202 m and 5.566 m, respectively, reduced by 60.448%. The average deviations of gully edge line are 3.271 m and 10.036 m, respectively, reduced by 67.406%. The identification rate and the position accuracy of gully edge line and gully head produced by the new method are significantly higher than that by the traditional method of interpreting remote sensing images only. The results of this study provide a new method for gully erosion research based on remote sensing images.

## 2. Methods

### 2.1. The Study Area

**Figure 1:**
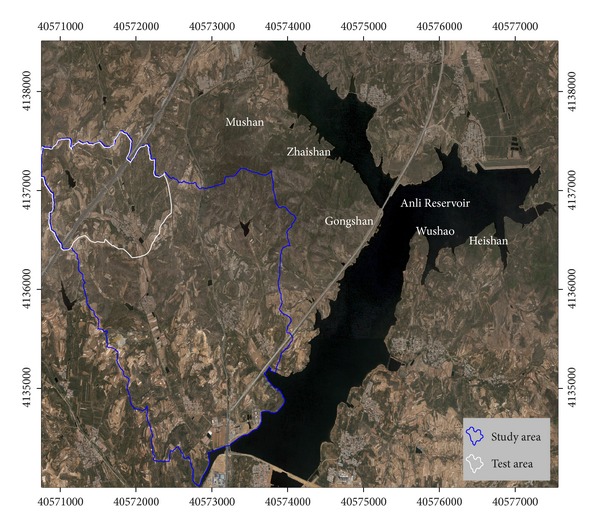
Location of the Qixia study area on a Quickbird true color image.

Qixia city is located in the hinterland of Shandong Peninsula, Shandong province, China, South Shore of Bohai Bay. The study area, a small watershed of Beiyanzikou (north latitude 37° 20′5 .47–37° 22′3 .17, east longitude 120° 47′55 .44–120° 50′10 .58), is located in northwestern suburb of Qixia city, about 3 km from Qixia city proper, with drainage area of 7158258.088 m^2^ (Figure 1). The area is hilly with altitude of between 130 and 180 m and an average elevation of 175 m. The area belongs to the warm temperate semi-humid monsoon climate, with annual average temperature of 11.3°C and annual rainfall of about 650 mm. Rainfall is concentrated in summer with July-August accounting for 57% of the annual precipitation, of which is mostly heavy rain and storm. Concentrated rainfall and interannual variability are the main factors causing soil erosion in the area. The main soil type in the study area is brown soil, accounting for 83.59% of the total area. The test region is located in the northwest corner of the study area with an area of 1540791.036 m^2^ (see Table 1). There are 155 and 52 gullies distributed in the study area and the test region, respectively, with gully thalweg length greater than 30 m and the average gully bottom wider than 5 m. Gullies in the study area are mainly aged hill slope gullies of “U” type, which develop into bank gullies and floor gullies at the same time. There are artificial terraces constructed on both sides of most gullies, which destroyed the original erosion landform. The lands between the gullies are mainly orchards and farmland with most steep slopes distributed with grass and shrubs; the bottom of the gullies are mainly distributed with grass, shrubs, and woodland.

### 2.2. Data Used

The data used in this paper is as shown in Table 1. The 5 m spatial resolution DEM data were acquired based on aerial stereo photogrammetry technology. The coordinate system of all data is unified into 1980 Xi'an Coordinate System.

### 2.3. Methodology

**Figure 2:**
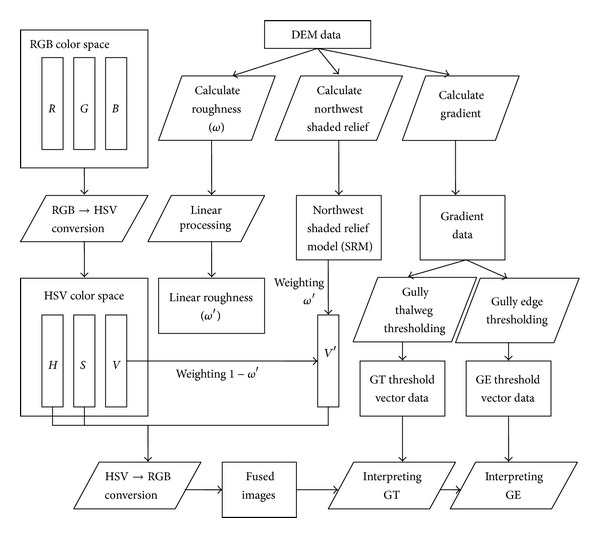
Schematic diagram of the interpreting gully edge from Quickbird image/Northwest SRM fused image.

Based on the study area's DEM data and elevation angle and azimuth of the solar according to the imaging time of the remote sensing image, the northwest illumination SRM data were built and then integrated into the remote sensing imagery on the basis of HSV color space conversion techniques to remove the FTPP of remote sensing images. Hence, precise identification of gully position might be made based on the fused image. Moreover, in combination with field measurements of gradient threshold values of gully thalweg and gully edge line, the gradient data were segmented by thresholding to obtain gully thalweg and edge line threshold vectors. Integrated with gully thalweg threshold vectors, edge line threshold vectors, fused image, and slope gradient data, gully thalweg and gully edge lines might be effectively interpreted. The detailed process is as shown in Figure 2.

#### 2.3.1. Image Processing

HSV (hue, saturation, value), also known as hexagonal pyramid model (Hexcone Model), is a kind of color space created by Smith according to the intuitive nature of color [22]. HSV color model parameters are hues (*H*), saturation (*S*), and value (*V*). The hues produced are in the range of 0 to 360 degrees (where red is 0 degrees, green is 120 degrees, and blue is 240 degrees) and saturation and value in the range 0 to 1 (floating-point). Value ranges from approximately 0 to 1 with higher numbers representing brighter colors [23].

First, the remote sensing imagery is transformed from RGB color space into HSV color space to obtain *H*, *S*, and *V* components. And simulated northwest illumination SRM is built according to solar elevation angle and azimuth at the imaging time and DEM data. Then linear standardization terrain roughness *ϖ*′ is taken as simulated northwest illumination data SRM weights and 1 − *ϖ*′ as *V* component weights to build a new color brightness value *V*′ based on their summation, which is retransformed to RGB color space (see Figure 2) with the combination of *H*, *S* components [24].

From the topographical point of view, roughness of the terrain can be defined as the degree of terrain ruggedness. Roughness of the terrain can be identified by the surface slope (change of elevation). The specific steps for the technique are as follows.

The linear standardization terrain roughness *ϖ*′ can be calculated:

(1) $$\begin{array}{c} \varpi^{\prime }=\frac{\varpi }{\max\left(\varpi \right)}, \end{array}$$

where max⁡(*ϖ*) is *ϖ*'s maximum value; *ϖ*′ is valued (0,1]; *ϖ* is terrain roughness that can be calculated by

(2) $$\begin{array}{c} \varpi =\frac{1}{\cos\left(\text{Slope\_rad}\right)}, \end{array}$$

where Slope_rad is slope gradient data with radians as the unit.

By formula (3) the amended color value component *V*′ can be calculated:

(3) $$\begin{array}{c} V^{\prime }=\varpi^{\prime }*\text{SRM}+\left(1-\varpi^{\prime }\right)*V, \end{array}$$

where *V*′ is the amended color brightness value; *ϖ*′ is linearized terrain roughness; SRM is simulated northwest illumination SRM data *V*′, and *H* and *S* components are retransformed from HSV color space into RGB color space to achieve the integration images of three-dimensional topographic information with spectral information of the remote sensing images.

The slope gradient of each pixel is calculated based on DEM data and the average gradient threshold values of gully thalweg and gully edge line were measured in field. The gully thalweg and edge line vectors were interpreted by binarization thresholding of the slope data based on gully thalweg and gully edge line gradient threshold values, respectively. And gully thalweg threshold vectors are amended precisely by using visual interpretation method based on the fused image of remote sensing imagery and topographic information. Thus precise interpretation of edge line can be made by using visual interpretation method in combination with amended gully thalweg vectors, fused image, and edge line threshold vectors.

#### 2.3.2. Accuracy Assessment

Inherent uncertainty of remote sensing data spatial scales and object spectrum and errors induced by the image processing, such as acquisition, resampling, transmission, interpretation, and classification, inevitably lead to the uncertainties in remote sensing imagery mapping. There are two types of map accuracy assessment: thematic and positional. Thematic accuracy deals with the labels or attributes of the features of a map and measures whether the mapped feature labels are different from the true feature label. Positional accuracy deals with the accuracy of the location of map features and measures how far a spatial feature on a map is from its true or reference location on the ground [25].

The error matrix has become a common mean to assess the thematic accuracy of remote sensing image-based classification [26–28]. In this study, gully class and non-gully class reference data are selected based on random sampling in the vicinity of the interpreted gully head and gully edge line. Then the confusion matrix is computed to assess the overall accuracy, producer accuracy and user accuracy with the overall accuracy as gully thematic accuracy.

Another discrete multivariate technique of use in accuracy assessment is called kappa [26–29]. Kappa coefficient represents the ratio of the reduced errors generated by evaluated classification to completely random classification.

The positional accuracy as the degree of compliance with which the coordinates of points determined from a map agree with the coordinates determined by survey or other independent means accepted as accurate [30]. Many factors lead to the positional accuracy of a map or georeferenced image such as the sensor lens may be distorted, or the aircraft carrying the sensor may suddenly tilt or yaw, changing the relationship of the sensor's image plane to the ground [29].

The deviation is the minimum nearest distance between interpreted gully edge line by using remote sensing images and field measuring points along gully edge. The average deviation *δ* is the quotient of the sum of absolute value of the minimum nearest distance calculated between the points and the interpreted gully edge line divided by the total number of points (formula (4)), which is used to assess overall positional accuracy of the interpreted gully edge line:

(4) $$\begin{array}{c} \delta =\frac{\left|\sum_{i}^{N}\gamma_{i}\right|}{N}, \end{array}$$

where *i* is field measuring point number, *γ*_*i*_ is the minimum distance between *i* measuring point and the nearest gully edge line, and *N* is the total number of field measuring points.

## 3. Results and Discussion

The Quickbird visible imagery is registered to the aerial orthographic DOM image (image-to-image registration) and is resampled to have a spatial resolution of 2.5 m by using the nearest neighbor method. The root-mean-square error (RMS errors) of the control points selected was 2.1344 pixels, exceeding 0.5 m, with large geometric registration error (see the reasons analyzed in the latter parts). The DEM data with spatial resolution of 5 m was resampled to 2.5 m in accordance with the Quickbird visible imagery.

**Figure 3:**
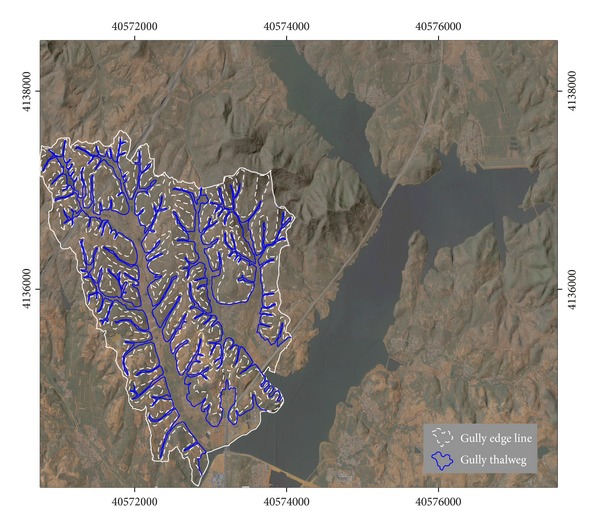
Results of fusing Quickbird image and Northwest SRM.

The simulated northwest SRM based on DEM data of the study area is calculated and integrated into the remote sensing images. Since the topographic information compliance with visual habits of human visual perception is integrated into the remote sensing images, the three-dimensional information of the gullies is clearly shown in the fused image (Figure 3).

During April 2008, field measurement of slope gradient along gully thalweg and gully edge line with 2.5 m spacing was conducted and obtained the slope gradient data of 2,208 gully thalweg measuring points and 5,286 gully edge line measuring points. Statistics show the gully thalweg average gradient was 7.411° with standard deviation of 2.000° and gully edge line average gradient was 13.103° with standard deviation of 4.631°.

Calculate slope gradient data of the study area using Slope program of Topographic Modeling tools of ENVI4.8 software [23] based on DEM data and take 7.411° and 13.103° as gully thalweg gradient threshold value and gully edge line gradient threshold value to binarizate the gradient data to obtain gully thalweg threshold vectors and gully edge line threshold vectors, respectively.

Combined with the fused imagery and gradient data, the gully thalweg threshold vectors were amended and 150 gullies with length greater than 30 m and bottom width greater than 5 m were interpreted ultimately, of which 46 gullies were interpreted in the test region. With comprehensive use of amended gully thalweg threshold vectors, fused image, gradient data, and gully edge threshold vectors, the edge lines of the 150 gullies were interpreted precisely.

**Figure 4:**
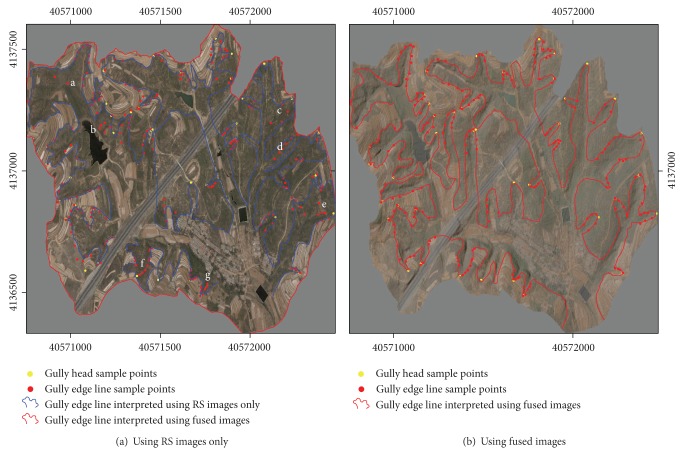
Gully edge lines interpreted and points field measured along gully head and gully edge.

A total of 52 gullies (see Figure 4) were interpreted based on Quickbird images only in the test region. Since there are less three-dimensional information and the FTPP in the original remote sensing images, it is difficult to identify gullies by using remote sensing images only. In addition, there was a lot of vegetation growing in the study area at the imaging time, in particular, in gullies of steeper terrain with low shrubbery growing across the gully areas, which increased the difficulty of interpreting gully edge line accurately.

There are 49 gullies distributed in the field survey test region (with gully bottom length greater than 30 m, bottom average width greater than 5 m). Reference points are selected in the vicinity of the gully head and around the gully edge line of the interpreted gullies to build gully class and non-gully class reference data. The confusion matrix was calculated.

The overall accuracy and user accuracy of gully interpreted using remote sensing images only are 61.207% and 52.632%, respectively. By using the fused image, overall accuracy and user accuracy are 97.414% and 100%, respectively. Since gully class reference sample points are randomly selected in the vicinity of the interpreted gully heads, they have certain statistical significance. 40 of the 52 gullies interpreted by interpretation using remote sensing image only are correctly interpreted and 12 are misinterpreted. Hence, overall accuracy and user accuracy are not high. The 46 gullies interpreted by using fused image are all correct by field verification, so the user accuracy can reach up to 100%.

Kappa coefficient of interpreted remote sensing only and fused image is 0.946 and 0.260, respectively, increased by 264.364%.

There is an obvious difference for the overall accuracy between the two methods because the fused image not only contains feature spectral information but also has simulated northwest illumination topographical information. Thus it can more accurately interpret gully topographic features and the interpretation omission accuracy of gully is 0 while interpretation error rate reached up to 18.367% by using remote sensing imagery only. In addition, since the adopted resolution of the DEM and remote sensing imagery is 2.5 m, gully bottom with an average width less than 5 m and gully edge line with an average gradient of less than 7.411° cannot effectively be interpreted.

In the assessment with uncertainty of sampled data properties, sample data quality has significant impact on the error matrix. The sample size and sampling schemes are two important factors affecting the quality of the reference data. Sample size includes sampling unit and the number of samples. On the one hand, in order to be statistically significant, the sample size should be as large as possible. On the other hand, the size of the sample should be as small as possible to reduce the cost. A large number of accuracy assessments have been conducted by using a single pixel as the sampling unit [29]. In the test, 116 sampling points were selected for both gully class (49 points) and non-gully class (67 points) to meet the requirement of at least 30 sampling points for each class [29]. A poor sampling scheme may lead to overestimation or underestimation of the accuracy. There are five common sampling schemes that have been applied for collecting reference data: (1) simple random sampling, (2) systematic sampling, (3) stratified random sampling, (4) cluster sampling, and (5) stratified, systematic, unaligned sampling [29]. Because the study objects are gullies, one single object, and we have some prior knowledge on the distribution of gullies, thus stratified random sampling method was used to randomly select reference data in the vicinity of gully head and outside of gully edge line of the interpreted gullies. Therefore, the sampling size and sampling scheme used in the test are reasonable and the calculated error matrix can indicate the interpretation accuracy.

The field measurements along gully head and gully edge line were conducted to form gully edge line measuring point data and to calculate the minimum nearest distance, maximum nearest distance, and average distance between the gully head and gully edge line interpreted by the two interpretation methods and field measuring points using the Near tool of ArcGIS Desktop 10.0 software. The results are as shown in Table 2.

Compared with the method of using remote sensing images only, the average positional deviation of gully head and edge line interpreted by using the fused image of topography and remote sensing images is 2.2 m and 3.271 m, respectively, increased by 60.448% and 67.406%, respectively. Statistics show that positional accuracy of gully head and gully edge line interpreted by using fused image of topography and remote sensing images is significantly higher than that of using remote sensing images only.

Spatial resampling (2.5 m) leads to spectral distortion of Quickbird optical images, and the spectral information of land between gullies covered with grass is easily confused with gully area. In addition, remote sensing two-dimensional images generally have FTPP [24, 31]; therefore, interpretation accuracy is low by using remote sensing images only. As shown in Figure 4(a), the average deviation between the interpreted gully edge lines and field measuring points is large by using simple remote sensing interpretation method.

**Figure 5:**
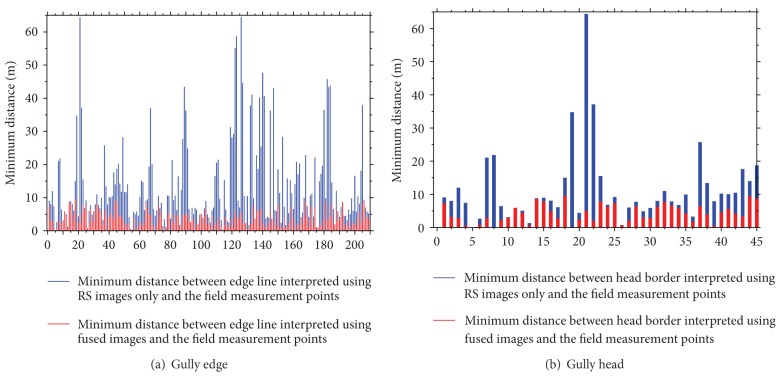
Minimum distance between points field measured and gully interpreted using RS images only and fused images.

Moreover, the results show that positional accuracy of gully edge line and gully head interpreted by using remote sensing images only is closely related to land use type between gullies. The position error is larger in areas between gullies where land use types are shrub fields or forest land while smaller in areas between gullies where there is farmland. Measuring points with deviation more than 20 m are mostly distributed in a, b, c, d, e, and f areas in Figure 4(a) where there is non-farmland (shrubs, grass, shelter, etc.). Therefore, it will be difficult to accurately extract the gully edge lines in these areas by using remote sensing spectral information alone.  Figure 4(b) is gully edge line map extracted based on the fused image of topography and remote sensing images. As topographic factor, such as simulated northwest illumination SRM, is merged with Quickbird optical satellite images, the positional accuracy gully edge line for non-farmland areas interpreted by using the fused image is significantly increased.  Figure 5 plots the detailed error between the two approaches.

In the test, the location of gully edge lines (including gully head) interpreted by using optical satellite images only and by using fused image of topography and optical remote sensing images is not completely consistent with the field measuring points. There are mainly five reasons. (1) As the mosaic horizontal coordinate precision of the DOM image used is 1.2 m and mosaic tolerance is 0.4 m, thus the coordinate error of DOM used for geometric correction is greater. (2) The controlled point root-mean-square error (RMS errors) is greater than 0.5 m in Quickbird image geometric register process; thus DOM data coordinate error and image registration error are the main errors of the study. (3) Because the sensor image plane is flat and the earth has relief such as hills and ravines, the impact of topography on remotely sensed imagery is the most important cause of positional error [29]. (4) Uncertainty of raster data (remote sensing image, DEM, slope data) resampling and other operational processing may also result in part of errors in the interpretation.

## 4. Conclusions

Of all measuring techniques for monitoring the initiation and development of gullies, remotely sensed image-based techniques have become one of the important means. However, due to the impact of factors such as spatial resolution scales, uncertainty of spectra information, and false topographic perception phenomenon (FTPP) in remote sensing imagery, it is difficult to achieve high precision interpretation of gully edge lines by using optical satellite imagery only. This paper, taking the small watershed of Beiyanzikou valley of Qixia city, Shandong province, as the study area and taking Quickbird remote sensing images as experimental data proposed a method of using terrain information with spectral information of remote sensing image fusion technique. The technique based on HSV conversion established new value components by summing up simulated northwest illumination shaded relief data (SRM) and *V* component (value) with linear surface roughness *ϖ*′ and 1 − *ϖ*′ as weighting, respectively. Then they were retransformed into RGB color space to achieve FTPP removed shaded relief data and were integrated into the remote sensing images to enhance and enrich terrain information in the image. Then field measurements of gully edge line and gully thalweg topographic slopes were conducted to obtain gully edge line and gully thalweg average gradient threshold values. We calculated the gradient based on DEM data and segmented the gully edge line and gully thalweg to obtain gully edge line and gully thalweg threshold vectors. Gully thalweg threshold vectors were amended to extract the gully thalweg with high precision based on fused image of topographic information and remote sensing data in combination with gully thalweg threshold vectors. Then gully edge line was interpreted based on the amended gully thalweg vectors, fused image, and gully line threshold vectors. To assess the accuracy of the method, a testing area was selected in the study area. Accuracy assessments of the gully interpreted by both the traditional simple interpreting using remote sensing image only and using fused image of topography and remote sensing imagery were performed through field spot measurements of gully head and gully edge line in the testing region by taking deviation, kappa coefficient, and overall accuracy of error matrix as the indices to evaluate the accuracy of the interpreted gully information (gully, gully head, and gully edge line). The results show that overall accuracies are 97.196% and 78.333%, respectively, increased by 24.080%; kappa coefficient is 0.946 and 0.260, respectively, increased by 264.364%; gully head average deviations are 2.202 m and 5.566 m, respectively, reduced by 60.448%; gully edge line average deviations are 3.271 m and 10.036 m, respectively, reduced by 67.406%. The test results indicate that, compared with traditional simple remote sensing image visual interpretation method, the accuracy of interpreting gullies and gully edge line by using the new method is significantly higher. This result provides a new approach to the study of gully erosion based on remote sensing images.

## Figures and Tables

**Table 1:** Basic information of the experiment data.

Data	Time (GMT)	Spatial resolution (m)	Explanation
Quickbird image	2004-5-15: 2:02:36	0.6	Visible light (red, green, and blue) Elevation: 59.687558° Azimuth 119.714057°
DEM	—	5	DEM raster data
RTK-GPS	2008-4	0.5	The level accuracy of dynamic measurement is 10 mm ± 1 ppm. The vertical accuracy of dynamic measurement is 20 mm ± 1 ppm
Aerial photographs (DOM)	2008-5	0.25	Visible light (red, green, and blue). Orthographical correction

**Table 2:** The positional errors of gully for the two approaches (unit: m).

Interpreting approach	Gully head			Gully edge line		
	Min	Max	Mean	Min	Max	Mean
Using RS only	0.012	48.669	5.566	0.014	59.506	10.036
Using fused image	0.002	6.315	2.202	0	9.583	3.271
